# Prediction of survival and outcomes with depth of response at 6 months in metastatic non-small-cell lung cancer patients treated with chemotherapy and immunotherapy combination: SPORE trial

**DOI:** 10.1016/j.esmoop.2025.106042

**Published:** 2026-01-29

**Authors:** F. Moinard-Butot, J.-B. Barbe-Richaud, J. Lasvergnas, J. Ancel, G. Justeau, H. Le Floch, L. Somme, C. Chouaid, O. Bylicki, R. Schott

**Affiliations:** 1 Medical Oncology department, Institut de Cancérologie de Strasbourg Europe (ICANS), Strasbourg, France; 2 Service de Pneumologie, Pneumology, Intercommunal Hospital, Créteil, France; 3 Pneumology Department, Reims Hospital, Reims, France; 4 Department of Respiratory and Sleep Medicine, Angers University Hospital, Angers, France; 5 Service de Pneumologie, Hôpital d’Instruction des Armées, Clamart, France

**Keywords:** NSCLC, metastatic, depth of response, overall survival, immunotherapy, chemotherapy, long-term response

## Abstract

**Background:**

Metastatic non-small-cell lung carcinoma (mNSCLC) first-line therapy is based on the combination of chemotherapy with immune checkpoint inhibitors (ICIs). Early assessment of long-term outcomes may be crucial to guide clinician’s decisions. Whether radiological depth of response (DpR) could be a surrogate of survival remains unsolved.

**Objectives:**

This study aims to evaluate the correlation between the DpR at 6 months and survival in patients presenting an mNSCLC treated with ICI chemotherapy in a first-line metastatic setting.

**Methods:**

This retrospective multicenter study included mNSCLC patients treated with ICI chemotherapy, still on first-line therapy at 6 months, with measurable disease. Patients were classified into four groups according to radiological assessment at 6 months of the beginning of first-line therapy using RECIST criteria 1.1: group 1: −100% to −60%, group 2: −59% to −30%, group 3: 0% to −29% and group 4: 0% and more. We evaluate the impact of DpR on survival with the Cox model using group 3 (0% to −29%) as the reference.

**Results:**

The analysis included 175 patients: (median age: 61 years, adenocarcinoma: 82.3%; brain metastasis: 25.7%, PD-L1 ≥ 1%: 56.0%. Patients were in groups 1, 2, 3 and 4 in 21.1%, 31.5%, 29.7% and 17.7% of cases, respectively. Median overall survival and progression-free survival were 35.0 months [95% confidence interval (CI), 31.0-NA], and 17.1 months (95% CI, 14.0-23.9 months), respectively. Patients in group 1 and 2 had a significantly lower risk of death compared with group 3 [Hazard ratio (HR) = 0.31, 95% CI, 0.14-0.68, *P* = 0.003, and HR = 0.53, 95% CI, 0.30-0.92, *P* = 0.025, respectively].

**Conclusions:**

Depth of response at 6 months according to RECIST 1.1 criteria for patients with mNSCLC still on first-line therapy is a predictor of overall survival.

## Highlights

• Early prediction of long-term efficacy of ICI chemotherapy combinations in metastatic NSCLC remains a challenge.
• Impact of early radiological depth of response on the efficacy of chemo-immunotherapy in metastatic NSCLC is poorly studied.
• Depth of RECIST 1.1 response at 6 months for mNSCLC patients still on first-line therapy appears to be a predictor of OS.

## Introduction

Lung cancer remains a world health challenge, being the first cause of cancer death, with a rising incidence (estimated 2.2 million new cancer cases). While tobacco exposure remains the major risk factor, non-small-cell lung cancer also occurs in nonsmokers, leading to a heterogenous landscape of disease.^1^ Metastatic non-small-cell lung carcinomas (mNSCLCs) with oncogenic drivers, thus with a drug targetable alteration (DTA), typically have a better prognosis and can be managed with targeted therapies, whereas lung cancers without such drivers often have a poorer prognosis and rely more on conventional treatments like chemotherapy and immune checkpoint inhibitors (ICIs).^2^^,^^3^ Currently, there are no predictive biomarkers for chemotherapy combined with ICIs in lung cancer.

ICIs have transformed the treatment landscape for mNSCLC without DTA. Randomized trials have successfully demonstrated significantly improved outcomes combining these ICIs with platinum-based chemotherapy. The KEYNOTE-189 trial reported a median overall survival (OS) of 22 months [95% confidence interval (CI) 19.5-24.5 months] and an objective response rate (ORR) of 47.6% for patients presenting a metastatic adenocarcinoma treated with pembrolizumab plus chemotherapy, compared with a median OS of 11.3 months (95% CI 8.7-15.1 months) for chemotherapy alone (*P* < 0.001).^2^^,^^4^ Concerning squamous cell carcinoma (SCC), the KEYNOTE-407 trial indicated a median OS of 15.9 months (95% CI 13.2 months-NA) and an ORR of 57.9% with combination therapy, compared with a median OS of 11.3 months (95% CI 9.5-14.8 months) for chemotherapy alone (*P* < 0.001).^3^

Correlation between radiological response and survival outcomes have previously been studied in mNSCLC populations treated with chemotherapy alone. The field remains controversial, in terms of ORR, as well as tumor shrinkage.^5^, ^6^, ^7^ Concerning targeted therapy, the recent data highly suggest a correlation between ORR, depth of response (DpR), progression-free survival (PFS), and OS.^8^, ^9^, ^10^ Under a combination of chemotherapy and ICI, retrospective series indicated that complete response (CR) and long-term response (LTR) were possible in a fraction of patients (2%-5%) with mNSCLC, whose OS largely exceed 2 years.^11^^,^^12^ Among the predictive factors of LTR, ORR and DpR on the combination chemotherapy and ICI seem to be crucial.^13^

We hypothesize that early imaging and evaluation of DpR, defined by a 30% threshold for ORR beyond RECIST will provide prognostic relevance in mNSCLC without DTA. We present the SPORE trial, a retrospective multicenter study, aiming to evaluate the relationship between the DpR according to RECIST 1.1 criteria at 6 months from the beginning of the combination chemo-immunotherapy in first-line settings and survival.

## Materials and methods

### Study design and patient population

This study is a retrospective analysis of patients with mNSCLC without drug targetable alterations treated with a combination of chemotherapy and ICI as first-line setting. The patient cohort was derived from a database of individuals treated at five centers (Institut de Cancérologie Strasbourg Europe, University Hospital of Créteil, University Hospital of Reims, University Hospital of Angers, Military Hospital of Toulon), from February 2019 to December 2021. Inclusion criteria included a histologically confirmed diagnosis of mNSCLC without DTA, availability of baseline imaging with at least one measurable lesion and follow-up imaging at 6 months, treatment as first-line setting of a specified chemo-immunotherapy regimen still ongoing at 6 months. Patients were excluded if they presented a progressive disease, died before 6 months after the initiation of the first-line combination therapy, or refused to participate.

### Tumor response assessment

Tumor DpR was determined directly comparing baseline imaging [computed tomography (CT) scan or positron emission tomography–computed tomography] with 6 months of treatment imaging. OS and PFS, both retrospectively assessed, were determined by the following tumor response categories: −100% to ≤ −60%, −59% to ≤ −30%, −29% to < 0%, 0% to progressive disease. Specifically, the category of −100% to < −60% correspond to Group 1, −59% to < −30% to Group 2, −29% to 0% to Group 3, and 0% or more to Group 4.

### Statistical analysis

The primary endpoint was to determine whether tumor shrinkage ≥30% (group 1, group 2) at 6 months could be a predictive factor for OS, compared with group 3 (−29% to 0%). OS was defined as the time from the start of treatment until death from any cause. Secondary endpoints included assessment of OS and PFS of the cohort and of the four groups. PFS was defined as the time from the start of treatment until the first documented progression (or the start of subsequent therapy in case of missing data) or death. Kaplan–Meier methods were used to estimate OS and PFS distributions in the entire cohort, then between groups of tumor response. Comparisons between groups were made using the log-rank test. Cox proportional hazards models were employed to evaluate the impact of tumor shrinkage on OS, using group 3 (−29% to 0%) as the reference, firstly in a univariate model, then adjusted for potential confounders (after exclusion of subjects with missing data). Validity of this model (i.e. proportional hazard assumption, independence of variable, number of variables) was verified. A comparison between baseline characteristics in the four groups was made using chi-square test, Fisher’s exact test when chi-square test was not applicable, and ANOVA. Statistical analyses were carried out using a significance level set at *P* < 0.05. All statistical analyses and plots were done with R software (version 4.3.2).

### Ethical considerations

The study was conducted in accordance with the Declaration of Helsinki and approved by the institutional review board of each participating center. Informed consent was obtained from all patients before their inclusion in the study (IRB 2024-02).

## Results

### Patient’s characteristics

A total of 175 patients with mNSCLC treated between February 2019 and December 2021 were included in the analysis. The median age was 61 years, with 64.6% of patients being male. Among these, 144 (82.3%) had an adenocarcinoma, and 25 (14.3%) harbored an SCC. The majority of mNSCLCs were *de novo* (80%). All patients received a combination of chemotherapy and ICI as first-line treatment. Full demographic and clinical features of the patient cohorts are described in Table 1.

**Table 1:** Demographic and clinical features of the cohort

Characteristics	No. of patients *N* = 175 (%)
Median age (range)	61 (56-68)
Sex – no. **(%)**	
Male	113 (64.6%)
Female	62 (35.4%)
ECOG – no. (%)	
0-1	172 (98.3%)
2-3	3 (1.7%)
Smoking status – no. (%)	
Smoker (current or former)	162 (92.6%)
Non smoker	7 (4%)
Unknown	6 (3.4%)
Histology – no. (%)	
Adenocarcinoma	144 (82.3%)
Squamous cell carcinoma	25 (14.3%)
Other **–** unknown	6 (3.4%)
PD-L1 status – no. (%)	
≤1%	76 (43.4%)
1-49%	52 (29.7%)
≥50%	46 (26.3%)
Unknown	1 (0.6%)
Brain metastasis at diagnosis – no. (%)	
Yes	45 (25.7%)
No	109 (62.3%)
Unknown	21 (12%)
Most common sites of metastasis – no. (%)	
Lymph node	109 (62.3%)
Lung	108 (61.7%)
Bone	54 (30.9%)
Number of metastasis – no. (%)	
1-5	104 (59.4%)
≥6	62 (35.4%)
Unknown	9 (5.2%)
Disease presentation – no. (%)	
*De novo* metastatic	140 (80%)
Metastatic relapse	27 (15.4%)
Unknown	8 (4.6%)

### Tumor response

The distribution of patients among tumor response categories at 6 months was as follows: 37 patients (21.1%) in group 1, 55 patients (31.5%) in group 2, 52 patients (29.7%) in group 3, and 31 patients (17.7%) in group 4. Characteristics of patients according to their group of radiological response are presented in Table 2.

**Table 2:** Demographic and clinical features of patients according to their group of radiological response

Characteristics	Group 1 *N* = 37 (21.1%)	Group 2 *N* = 55 (31.5%)	Group 3 *N* = 52 (29.7%)	Group 4 *N* = 31 (17.7%)	*P* value∗
Median age (IQR)	59 (54-66)	61 (57-68)	63 (58-68)	61 (56-69)	0.65
Sex – no. (%)					
Male	22 (59.5%)	30 (54.5%)	36 (69.2%)	25 (80.6%)	0.08
Female	15 (40.5%)	25 (45.5%)	16 (30.8%)	6 (19.4%)	
Histology – no. (%)					
Adenocarcinoma	29 (78.4%)	46 (83.6%)	48 (92.3%)	21 (67.7%)	0.06
Squamous cell carcinoma	5 (13.5%)	9 (16.4%)	3 (5.8%)	8 (25.8%)	
Unknown	3 (8.1%)		1 (1.9%)	2 (6.5%)	
PD-L1 status – no. (%)					
≤1%	12 (33.3%)	23 (41.8%)	27 (51.9%)	14 (45.2%)	
1-49%	8 (22.2%)	16 (29.1%)	19 (36.6%)	9 (29%)	0.06
≥50%	16 (44.4%)	16 (29.1%)	6 (11.5%)	8 (25.8%)	
Unknown	1 (2.7%)				
Brain metastasis at diagnosis – no. (%)					
Yes	9 (24.3%)	17 (30.9%)	12 (23.1%)	7 (22.6%)	
No	23 (62.2%)	27 (49.1%)	36 (69.2%)	23 (74.2%)	0.42
Unknown	5 (13.5%)	11 (20%)	4 (7.7%)	1 (3.2%)	
Number of metastasis – no. (%)					
1-5	20 (54.1%)	38 (69.1%)	32 (61.5%)	14 (45.2%)	
≥6	16 (43.2%)	16 (29.1%)	15 (28.9%)	15 (48.4%)	0.15
Unknown	1 (2.7%)	1 (1.8%)	5 (9.6%)	2 (6.4%)	
Disease Presentation – no. (%)					
*De novo* metastatic	33 (89.2%)	43 (78.2%)	40 (76.9%)	24 (77.4%)	
Metastatic relapse	2 (5.4%)	8 (14.5%)	11 (21.1%)	6 (19.4%)	0.21
Unknown	2 (5.4%)	4 (7.3%)	1 (1.9%)	1 (3.2%)	

∗ *P* value of the statistical test comparing characteristics between the four groups, using ANOVA for ‘Age’, Fisher’s exact test for ‘Histology’ and ‘Disease Presentation’ and Chi-square test for ‘Sex’, ‘PD-L1 status’, ‘Brain metastasis at diagnosis’, ‘Number of Metastasis’.

The median time between the baseline and 6-month radiological assessment was 6.6 months (range: 5.8-7.5 months). One hundred and ten patients (62.9%) were evaluated using the same modality of imaging at baseline and at 6 months, whereas 65 patients (37.1%) were evaluated using a different type of imaging (Supplementary Table S1, available at https://doi.org/10.1016/j.esmoop.2025.106042).

### Overall survival and progression-free survival

**Figure 1:**
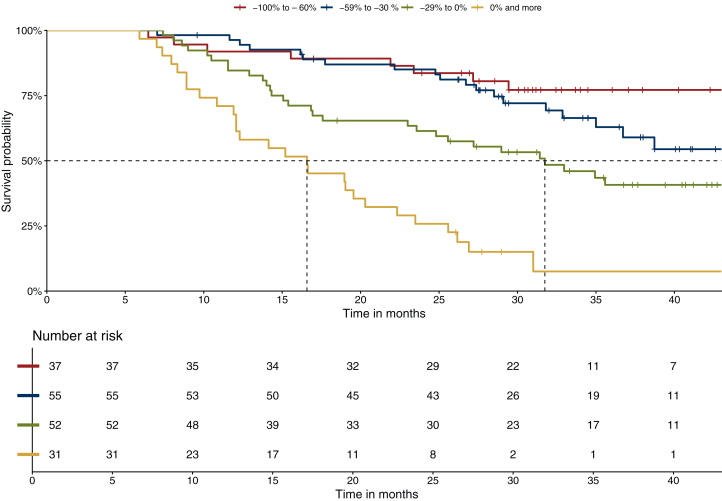
**Overall survival according to depth of response at 6 months.** Group 1 (−100% to −60%), red curve, median OS = not reached. Group 2 (−59% to −30%): blue curve, median OS = 45.5 months (95% CI 35.0 months-NA). Group 3 (0% to −29%): green curve, median OS = 31.7 months (95% CI 23.6 months-NA). Group 4 (0% and more): orange curve, median OS = 16.6 months (95% CI 12.1-23.5 months).

At data cut-off (15 June 2024), 86 patients died (49.1%), with 8 in group 1 (21.6%), 20 in group 2 (36.4%), 31 in group 3 (59.6%) and 27 in group 4 (87.1%). Median follow-up (initiation of the first-line immunochemotherapy to death or censorship) was 27.7 months. Median OS for the entire cohort was 35.0 months (95% CI 31 months-NA) (Supplementary Figure S1, available at https://doi.org/10.1016/j.esmoop.2025.106042). Median OS in group 1 was not achieved. Median OS in group 2 was 45.5 months (95% CI 35.0 months-NA), while median OS in group 3 was 31.7 months (95% CI 23.6 months-NA), and 16.6 months (95% CI 12.1-23.5 months) for group 4. (Figure 1).

The median PFS for the entire cohort was 17.1 months (95% CI 14.0-23.9 months) (Supplementary Figure S2, available at https://doi.org/10.1016/j.esmoop.2025.106042). Median PFS in group 1 was not reached. Patients in group 2 had a median PFS of 26.7 months (95% CI 22.2 months-NA). For patients in group 3 the median PFS was 13.7 months (95% CI 12.0-20.7 months), and 5.7 months (95% CI 5.5-8.0 months) for group 4. (Supplementary Figure S3, available at https://doi.org/10.1016/j.esmoop.2025.106042).

### Statistical analysis

**Figure 2:**
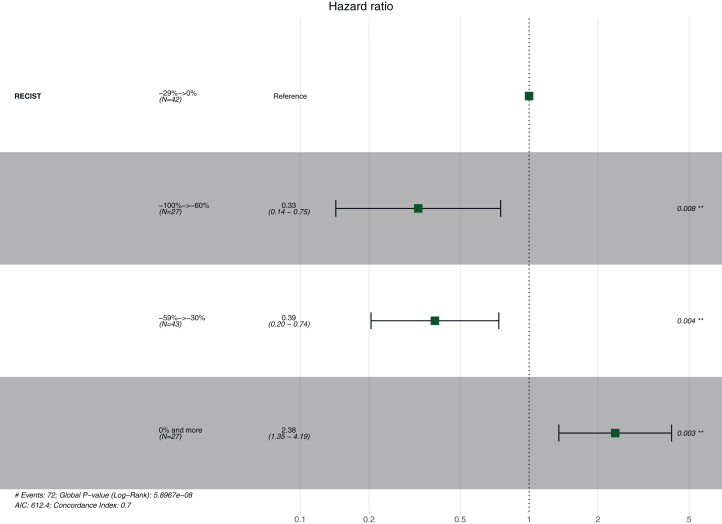
**Univariate Cox Regression Model evaluating the association between survival and tumor shrinkage at 6 months.** Forest plot showing hazard ratio and 95% confidence intervals for overall survival according to depth of radiological responss at 6 months. ∗∗Refers to the reference group (group 3, -29% to 0%), used as the comparator in the Cox regression model.

The log-rank test showed significant differences in OS and PFS among the different tumor response categories (*P* < 0.001). The Cox proportional hazards model confirmed that greater tumor shrinkage (group 1, group 2) was associated with improved OS [hazard ratio (HR) = 0.31, 95% CI 0.14-0.68, *P* = 0.003, and HR = 0.53, 95% CI 0.30-0.92, *P* = 0.025, respectively] compared with group 3 (Figure 2). Multivariate analysis adjusting for factors (histological subtype, PD-L1 score, presence of brain metastasis at baseline, and number of metastases at baseline) confirmed this association (Table 3).

**Table 3:** Results from multivariate Cox regression model, examining the association between survival and radiological response using RECIST 1.1 criteria at 6 months

	Hazard ratio (95%, CI)	Adjusted Hazard ratio^a^ (95%, CI)
Group 1: −100% to −60%	0.31 (0.14-0.68)	0.28 (0.12-0.67)
Group 2: −30% to −59%	0.53 (0.30-0.92)	0.32 (0.16-0.62)
Group 3: 0 to −29%	*Ref*	*Ref*
Group 4: 0% and more	2.80 (1.65-4.78)	2.14 (1.16-3.97)

a Adjusted based on histological subtype, PD-L1 score, presence of brain metastasis at baseline, and number of metastasis at baseline. Analysis conducted on 139 patients.

## Discussion

This trial confirms that achieving a CR or very good partial response (PR) (>60%) on combination chemo-immunotherapy is significantly associated with improved OS and PFS in mNSCLC. These results follow on from the pivotal phase III trials as KEYNOTE-189 and IMpower150, which respectively demonstrated substantial survival benefits with pembrolizumab and atezolizumab associated with chemotherapy in a first-line setting.^2^^,^^14^

The prognostic value of tumor response in mNSCLC is particularly noteworthy, with a clear and statistical link between DpR and survival. In our study, patients who achieved CR or very good PR (>60%) did not reach the median OS, for patients with good response (−30% to −59%), median OS was 45.6 months, compared with 31.8 months for PR (0% to −29%) and 16 months for progressive disease. These results align with previous research indicating that DpR is associated with longer survival.^5^^,^^15^ Apart from DpR, prolonged response is also correlated with extended survival, as is depicted in the 5-year update outcomes of CheckMate-017 and CheckMate-057 evaluating nivolumab, an ICI, in advanced lines in mNSCLC.^16^

Association between radiological response and survival has been demonstrated among metastatic renal cell carcinoma treated with ICI and vascular endothelial growth factor inhibitor in an analysis of patients enrolled in the CLEAR trial. Patients with CR or more than 75% of depth of response at 6 months had a survival >90% at 2 years.^17^ Similar conclusions seem to be outlined in metastatic melanoma,^18^ gastric cancer,^19^ pancreatic carcinoma^20^^,^ and colorectal cancer.^21^

The observed survival benefits linked with achieving CR and good PR emphasize the importance of striving for maximal tumor response in mNSCLC. These findings support the current clinical practice of using combination therapy to enhance treatment efficacy and improve patient outcomes. IMpower 150 evaluated the benefit of adding bevacizumab, a vascular endothelial growth factor inhibitor, to carboplatin, paclitaxel and atezolizumab, a PD-L1 inhibitor, in first-line metastatic setting (ABCP regimen) for patients presenting a non-squamous cell lung carcinoma. The quadruplet demonstrated an ORR of 69.3% (95% CI 61.3% to 76.5%) with a median OS of 19.2 months (95% CI, 17.0-23.8 months) at the price of increased toxicity.^14^ The KEYNOTE-189 and KEYNOTE-407 trials demonstrated that combining pembrolizumab with chemotherapy improved outcomes, with an ORR of 47.6% for metastatic adenocarcinoma, and an ORR of 57.9% for squamous cell carcinoma. This combination also demonstrated its efficacy among mNSCLC harboring driver mutation after progression on targeted therapy, with an ORR of 69.5%, but no difference in OS when compared with the control arm.^22^ ABCP also recently demonstrated its superiority in metastatic cervical cancer with improved PFS and OS, thus making the quadruplet regimen the new first-line standard of care.^23^ In daily practice, ABCP regimen is not the gold standard in France for mNSCLC, mainly because of increased toxicity. Feasibility of this quadruplet regimen is well established through the different clinical trials, however, as well as in recurrent or metastatic cervical cancer. With its high response rate and improved survival, ABCP regimen is attractive to achieve more DpR, thus leading to prolonged survival.

The 6-month landmark DpR seems to highly correlate with long term outcome, enhancing the possibility of early de-escalation for selective patients achieving very good radiological response. For a substantial group of patients who achieve long response, discontinuation of ICIs remains a key question, particularly given the lack of overall survival benefit associated with continuing treatment beyond 2 years.^24^^,^^25^ Retrospective studies highlight the possibility of early interruption without impact on long term survival.^26^ The same paradigm applied to melanoma,^27^ with ongoing prospective trials evaluating the safety of early interruption of ICI for patients presenting PR or CR.^28^^,^^29^

Because of differential outcomes, identifying patients at diagnosis who may present an LTR to ICI remains fundamental. The predictive value of PD-L1 expression for LTR remains controversial,^30^^,^^31^ whereas high tumor mutational burden may represent a predictive factor.^32^ Among clinical features, radiological response is unanimous.^13^^,^^15^^,^^31^^,^^32^ Our results reinforce the need for ongoing research to explore biomarkers that can predict which patients are most likely to achieve deep responses to combination therapies, thereby personalizing treatment approaches and optimizing clinical outcomes.

While our study provides valuable insights, several limitations should be noted. The retrospective nature of the analysis, coupled with potential selection biases inherent to clinical trial data, may affect the generalizability of our findings; even multi centre the number of centers remain limited. Furthermore, the 6 months landmark led to the exclusion of patients presenting a rapid progressive disease (<6 months) under combination therapy with a poor outcome, leading to the median OS of 35.0 months and median PFS of 17.1 months being observed, an outcome far superior compared with the outcomes reported in the PRINCEPS trials.^2^^,^^4^^,^^14^ It is worth noting that one-third of the patients were evaluated using a different type of imaging, and half of them with positron emission tomography–computed tomography. RECIST 1.1 criteria seem to be feasible using the CT from PET-Scans, however, largely dependent on the CT image quality.^33^ Finally, the majority of patients (144, 82.3%) had lung adenocarcinoma, and the analysis does not formally allow us to extrapolate the results to squamous NSCLC patients. Although we used a landmark analysis to address response bias, further validation studies are needed to confirm our results and refine predictive markers for treatment response.

Future research should focus on prospective trials to validate these findings and investigate the underlying mechanisms driving differential responses. Additionally, advancements in imaging techniques and the integration of functional imaging could enhance our ability to assess treatment responses more accurately and predict long-term outcomes, with a possibility of early de-escalation therapy for a subset of patients.

### Conclusion

Our study highlights the significant prognostic value of early tumor response in mNSCLC treated with first-line chemotherapy and ICI: DpR at 6 months forecast survival and outcome. The correlation between deep responses and improved survival underscores the efficacy of combination therapy in managing metastatic disease. Increasing DpR with intensive regimen followed with early de-escalation for selected patients should be explored. Continued research and prospective validation are essential to further optimize treatment strategies and improve patient outcomes in mNSCLC.
