# Supplementary material for: Prediction of survival and outcomes with depth of response at 6 months in metastatic non-small-cell lung cancer patients treated with chemotherapy and immunotherapy combination: SPORE trial
Source: ESMO Open. 2026 Jan 29;11(2):106042. doi: 10.1016/j.esmoop.2025.106042 (PMC12874283; doi:10.1016/j.esmoop.2025.106042)
Supplement: Supplementary Data [file mmc1.docx]

**Supplementary appendix**

**Contents :**

Page 2 **Supplementary Table 1 : Radiological assessment**

Page 3 **Supplementary Figure 1 : Overall Survival of the entire cohort**

Page 4 **Supplementary Figure 2 : Progression Free Survival of the entire cohort**

Page 5 **Supplementary Figure 3 : Progression Free Survival according to depth of response at 6 months**

**Table 1 : Radiological assessment**

| Modality  Of Imaging  Time | **CT Scan** | **PET-CT** | **Unknown** |
| --- | --- | --- | --- |
| **Baseline** | 89 (50.9%) | 86 (49.1%) | 0 (0%) |
| **At 6 month** | 139 (79.4%) | 34 (19.4%) | 2 (1.2%) |

**Figure 1 : Overall Survival of the entire cohort**

**
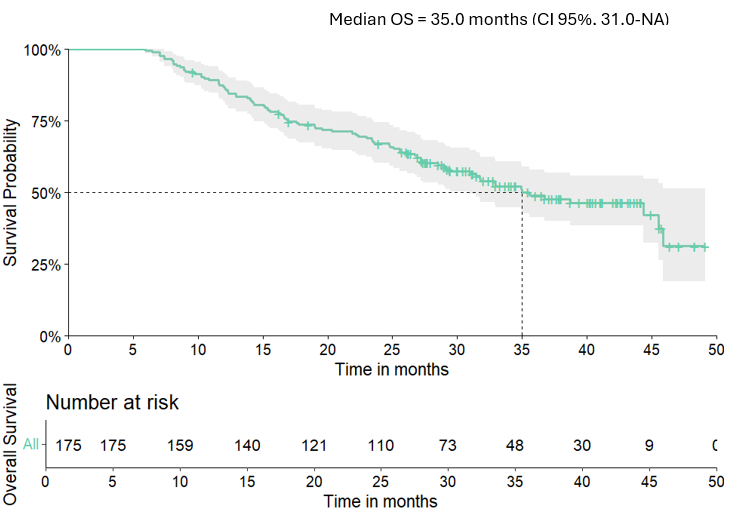
**

**Figure 2 : Progression Free Survival of the entire cohort**
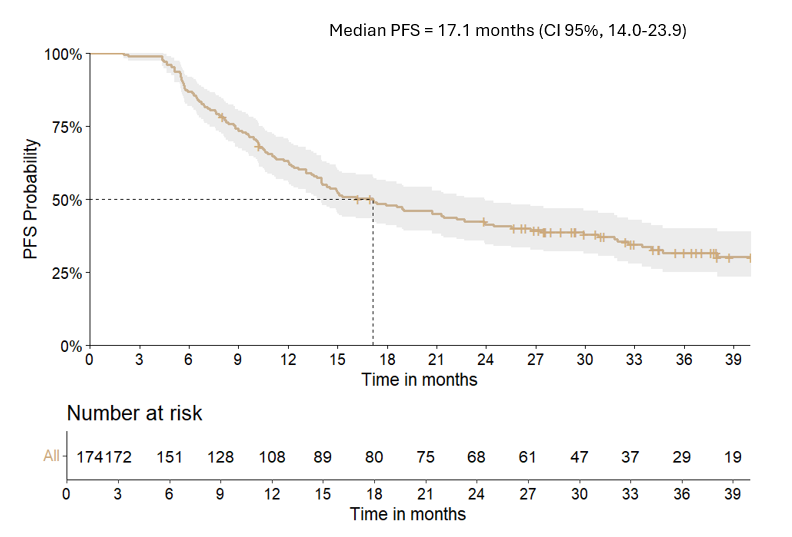


**Figure 3 : Progression Free Survival according to depth of response at 6 months**


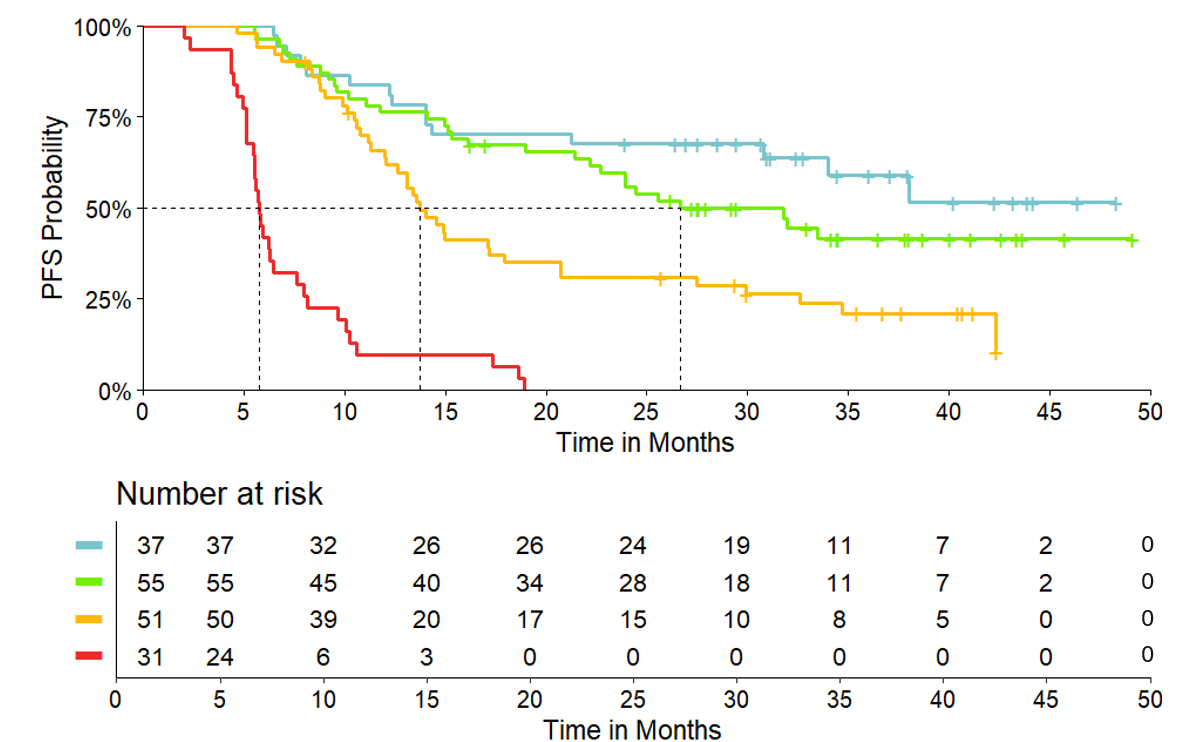


Group 1 (-100% to -60%), Blue Curve, Median PFS = not reached

Group 2 (-59% to -30%) : Green Curve, Median PFS = 26.7 months (CI95%, 22.2-NA).

Group 3 (0% to -29%) : Orange Curve, Median PFS = 13.7 months (CI95%, 12.0-20.7)

Group 4 (0% and more) : Red Curve, Median PFS = 5.7 months (CI95%, 5.5-8.0)
